# Glycation abolishes the cardioprotective effects of albumin during ex vivo ischemia‐reperfusion

**DOI:** 10.14814/phy2.13107

**Published:** 2017-01-26

**Authors:** Rudo F. Mapanga, Danzil E. Joseph, Marco Saieva, Florence Boyer, Philippe Rondeau, Emmanuel Bourdon, M. Faadiel Essop

**Affiliations:** ^1^ Cardio‐Metabolic Research Group (CMRG)Department of Physiological SciencesStellenbosch UniversityStellenboschSouth Africa; ^2^ Inserm UMR 1188 Diabète athérothrombose Thérapies Réunion Océan Indien (DéTROI) Université de La RéunionPlateforme CYROISaint Denis de La RéunionFrance

**Keywords:** Albumin, cardiac dysfunction, glycation, hyperglycemia, ischemia‐reperfusion, oxidative stress

## Abstract

Hyperglycemia‐induced oxidative stress plays a key role in the onset/progression of cardiovascular diseases. For example, it can trigger formation of advanced glycation end (AGE) products with ischemia‐reperfusion performed under hyperglycemic conditions. For this study, we hypothesized that albumin modified by glycation loses its unique cardioprotective properties in the setting of ischemia‐reperfusion under high glucose conditions. Here, ex vivo rat heart perfusions were performed under simulated normo‐ and hyperglycemic conditions, that is Krebs‐Henseleit buffer containing 11 mmol/L and 33 mmol/L glucose, respectively, ± normal or glycated albumin preparations. The perfusion protocol consisted of a 60 min stabilization step that was followed by 20 min of global ischemia and 60 min reperfusion. Additional experiments were completed to determine infarct sizes in response to 20 min regional ischemia and 120 min reperfusion. At the end of perfusions, heart tissues were isolated and evaluated for activation of the AGE pathway, oxidative stress, and apoptosis. Our data reveal that native bovine serum albumin treatment elicited cardioprotection (improved functional recovery, decreased infarct sizes) under high glucose conditions together with enhanced myocardial antioxidant capacity. However, such protective features are lost with glycation where hearts displayed increased infarct sizes and poor functional recovery versus native albumin treatments. Myocardial antioxidant capacity was also lowered together with activation of the intracellular AGE pathway. These data therefore show that although albumin acts as a cardioprotective agent during ischemia‐reperfusion, it loses its cardioprotective and antioxidant properties when modified by glycation.

## Introduction

Cardiovascular diseases (CVD) due to hyperglycemia are a leading cause of mortality (Mulnier et al. 2006). This poses a significant health burden as the global prevalence of diabetes is surging (Mbanya et al. 2010). There is a growing interest to better understand the association between hyperglycemia and CVD, for example, acute myocardial infarction (AMI) (Soedamah‐Muthu et al. 2006; Gardner 2014; Martín‐timón et al. 2014). There is a robust link between chronic hyperglycemia and macrovascular complications (Marcovecchio et al. 2011), with AMI a major factor leading to higher deaths within the diabetic context (Kannel et al. 1974; Diabetes Control and Complications Trial Research Group, 1993; United Kingdom Prospective Diabetes Study, 1998). Damaging effects also occur in the acute setting, with stress‐induced hyperglycemia linked to in‐hospital deaths, cardiogenic shock, and congestive heart failure (Capes et al. 2000; Sleiman et al. 2008; Marfella et al. 2009). Moreover, several reasons, for example, undiagnosed diabetes, impaired glucose tolerance (Norhammar et al. 2002; Sawin and Shaughnessy 2010), and stress‐mediated responses (Capes et al. 2000; Marfella et al. 2009) are put forward to explain the onset of hyperglycemia in persons that do not suffer from diabetes.

With both acute and chronic hyperglycemia, there is increased reactive oxygen species (ROS) production that activates similar metabolic and signaling pathways that mediate cardiac tissue damage (Marcovecchio et al. 2011; Mapanga and Essop 2016). Moreover, hyperglycemia‐induced oxidative stress originates from various processes such as excessive production of oxygen radicals from glucose autoxidation and mitochondrial respiration, glycoxidized proteins, and glycoxidation of antioxidative proteins (Li et al. 2007; Folli et al. 2011; Mapanga and Essop 2016). Collective data from biochemical, animal, and epidemiological studies support the hypothesis that glycoxidative modification of circulating proteins plays a pivotal and causative role in the pathogenesis of cardio‐metabolic diseases (Guerin‐Dubourg et al. 2012; Essop 2016).

Albumin is the most abundant serum protein with a wide range of biological properties (normal concentrations in range: 35–50 g/L) and is also susceptible to glycoxidative modification that may alter its structure and function (Francis 2010). Albumin is a highly soluble protein that contains 585 amino acids with a molecular weight of 66 kDa (Roche et al. 2008). This allows it to exert beneficial effects with the onset of various pathophysiologic conditions, for example, albumin can protect against ischemia‐reperfusion injury (Watts and Maiorano 1999; Robinet et al. 2007). There is ample evidence suggesting that albumin exerts significant antioxidant activity (Roche et al. 2008) and that it represents the predominant circulating antioxidant molecule that is therefore exposed to continuous stress (Phillips et al. 1989; Luoma et al. 1995). For example, hyperglycemia results in albumin glycation by the nonenzymatic attachment of glucose molecules to free primary amine residues that eventually leads to the formation of advanced glycation end products (AGE) [reviewed in Mapanga & Essop (2016)]. The formation and accumulation of AGEs constitute a characteristic feature of cardio‐metabolic complications; an effect they exert directly on the cells (Mullarkey and Edelstein 1990) or via interaction with the receptor for advanced glycation end products (RAGE) (Neeper et al. 1992; Yan et al. 1994). Binding of such ligands to RAGE induces intracellular signal transduction, leading to the activation of several proinflammatory pathways (Bierhaus et al. 2001; Zieman and Kass 2004; Yonekura et al. 2005). Thus, RAGE acts, at least in part, as an activator of carbonyl stress and is implicated in various cardio‐metabolic disorders (Sell et al. 1992).

Epidemiological studies established an inverse relationship between serum albumin levels and mortality risk; this is also the case with CVD (Phillips et al. 1989; Luoma et al. 1995). We previously reported on the damaging effects of increased myocardial AGE formation with ex vivo ischemia‐reperfusion performed under simulated hyperglycemic conditions (Mapanga et al. 2014). As glycated albumin can result in cardiac dysfunction during ischemia‐reperfusion by interfering with the beta 1 adrenoreceptor (Robinet et al. 2007), we hypothesized that albumin glycation results in loss of its cardioprotective properties during ischemia‐reperfusion under conditions of simulated acute hyperglycemia.

## Materials and Methods

### Synthesis and validation of glycated bovine serum albumin (BSA_gly_)

For this part, we tested the glycation on both BSA and human serum albumin (HSA). However, no subsequent experiments were carried out using HSA. BSA (Sigma‐Aldrich, St. Louis MO) was prepared separately at 40 g/L in 1× PBS and filtered. The solutions were subsequently divided into two components, the first native part and the second part which was glycated using 40% methyglyoxal solution at a final concentration of 10 mmol/L, filtered and dialysed. The native BSA was used for control experiments, whereas the glycated BSA was subsequently quantified (to final concentration of 0.01 mmol/L in the perfusate). We evaluated the success of BSA or HSA glycation by measuring fructosamine (product of glycation) levels using the method developed by Johnson et al. (1983) by employing the nitroblue tetrazolium reagent. The protocol was described in a previous study from our group (Guerin‐Dubourg et al. 2012). Results are expressed as mmol/L of 1‐deoxy‐1‐morpholinofructose which is a synthetic ketoamine used as a primary standard. Furthermore, the levels of free amine groups were also quantified using the 2, 4, 6‐trinitrobenzenesulfonic acid assay (Snyder & Sobocinski, 1975). This method was described in detail in a previous study by our group (Rondeau et al., 2010). Various concentrations of L‐glycine (10–200 nmol) were used to define the standard curve. In addition, molecular weights of both glycoxidized and non‐glycoxidized samples were determined by mass spectrometry.

### Animals and ethics statement

The Animal Ethics Committee of Stellenbosch University approved this study and rats were treated in accordance with the Guide for the Care and Use of Laboratory Animals of the National Academy of Sciences (NIH publication No. 85‐23, revised 1996).

### Ex vivo perfusion protocol

Male Wistar rats (180–220 gr) were employed for all experiments and after anesthetized (pentobarbitone, 100 mg/kg i.p.) hearts were swiftly excised for perfusion studies. Here, we used a modified Langendorff model (Mapanga et al. 2011) with Krebs‐Henseleit buffer containing (in mmol/L) 11 Glucose, 118 NaCl, 4.7 KCl, 1.2 MgSO_4_.7H_2_O, 2.5 CaCl_2_.2H_2_O, 1.2 KH_2_PO_4_, 25 NaHCO_3_ was equilibrated with 95% O_2_‐5% CO_2_ (37°C, pH 7.4) at a constant pressure (100 cm of H_2_O). The hearts were permitted to beat at its natural rate and buffer was not recirculated. A latex balloon (attached to pressure transducer) (Stratham MLT 0380/D, ADInstruments Inc, Bella Vista NSW, Australia) was put into the left ventricle. This was carefully executed via the mitral valve and the balloon thereafter inflated to generate systolic and diastolic pressures of 80–120 and 4–12 mm Hg, respectively. The perfusion pressure was maintained at approximately 70–80 mmHg during the stabilization period. We also employed a heated water jacket to ensure that the temperature for perfused hearts was consistently maintained at 37°C for the entire duration of the experimental protocol.

Hearts were randomly distributed into six experimental groups: control (11 mmol/L glucose) ± native BSA or BSA_gly_; and high glucose (33 mmol/L) ± BSA or BSA_gly_ (*n* = 6 rats per group). As ex vivo Langendorff perfusions are normally performed with 11 mmol/L glucose, we simulated acute hyperglycemia by employing a 33 mmol/L glucose concentration, a threefold increase. Perfusing with glycated albumin in the high glucose groups was included to elucidate its effects *per se* in control groups and in combination with high glucose.

### Ex vivo global ischemia and reperfusion during simulated acute hyperglycemia

Here, a 60 min stabilization period was followed by 20 min of global ischemia and 60 min reperfusion. The following functional parameters were evaluated: heart rate (HR), left ventricular developed pressure (LVDP), end diastolic pressure (EDP), rate‐pressure product (RPP = HR × LVDP), and coronary flow. In addition, we also determined the postischemic percentage recovery for LVDP, velocity of contraction (dP/dt), and RPP. In this case, we expressed reperfusion values as a percentage of preischemic data points. The coronary flow was also evaluated by pooling perfusion effluent at specific times. After the reperfusion stage, left and right ventricular tissues were rapidly collected by freeze‐clamping and subsequently stored at −80°C for additional analyses.

### Ex vivo regional ischemia and reperfusion during simulated acute hyperglycemia

To strengthen the contractile functional data, the effects of BSA and BSA_gly_ on infarct size were also assessed (20 min regional ischemia, 120 min reperfusion); infarct size and area‐at‐risk were determined as described before (Mapanga et al. 2012, 2014). Briefly, a 3/0 silk suture was placed around the proximal portion of the left anterior descending coronary artery and suture ends passed through a plastic tube to form a snare. For induction of regional ischemia, the snare was occluded and after 20 min, it was released to initiate reperfusion. The efficacy of ischemia was confirmed by regional cyanosis and a substantial decrease in coronary flow. After completion of each regional ischemia and reperfusion experiment, the snare was retightened and 2.5% Evans blue dye (in Krebs buffer) was perfused through hearts for identification of the area at risk of ischemia. Hearts were subsequently removed from the Langendorff apparatus, blotted dry, suspended (using suture) within 50 mL plastic tubes, and frozen at −20°C for 3 days. Thereafter, frozen hearts were sliced into 2 mm transverse sections and incubated with 1% 2,3,5‐triphenyl tetrazolium chloride (TTC) in phosphate‐buffered saline for 20 min at 37°C to distinguish noninfarcted (stained) from infarcted (nonstained) tissues. The area that was not stained with Evans Blue was defined as the area at risk (AAR). The area that displayed neither blue nor red was defined as the infarct site. Slices were then fixed in 10% formalin for 24 h at room temperature before being placed between glass plates for scanning for preparation of the phosphate‐buffered saline). We determined infarct size and the AAR by employing Image J software (v1.46p, National Institutes of Health, USA). For this study, the infarct size is expressed as a percentage of the AAR.

### Measurement of tissue AGE and fructosamine‐3‐kinase (FN3K) concentrations

We employed commercial ELISA kits to assess the myocardial concentrations of AGE (Cell Biolabs, San Diego CA) and FN3K (Cusabio, Baltimore MD) in heart tissue lysates, according to the manufacturer's instructions.

### Western blot analysis

After global ischemia, we isolated left and right ventricular tissues for homogenization and thereafter protein extraction and immunoblotting were performed with representative markers for: apoptosis repressor with caspase recruitment domain (ARC; Sigma‐Aldrich, St. Louis MO); AGEs, RAGE; superoxide dismutase 1 and 2 (SOD1, SOD2) (all from Abcam, Cambridge MA). Standard densitometric analysis was employed to quantify blotting data and we used β‐actin (Cell Signaling, Danvers MA) as a control for loading (Mapanga et al., 2012).

### Statistical analysis

All the data obtained were nonparametric, therefore, statistical analysis was performed by the Mann‐Whitney t‐test to compare between two groups. Kruskal‐Wallis one‐way analysis of variance (ANOVA) followed by the Dunns *post hoc* test (GraphPad Prism v5, San Diego CA) was used to compare means of three or more groups. Data are presented as mean ± standard error of mean (SEM) and values were considered significant when *P* < 0.05.

## Results

### Validation of BSA glycation

**Figure 1:**
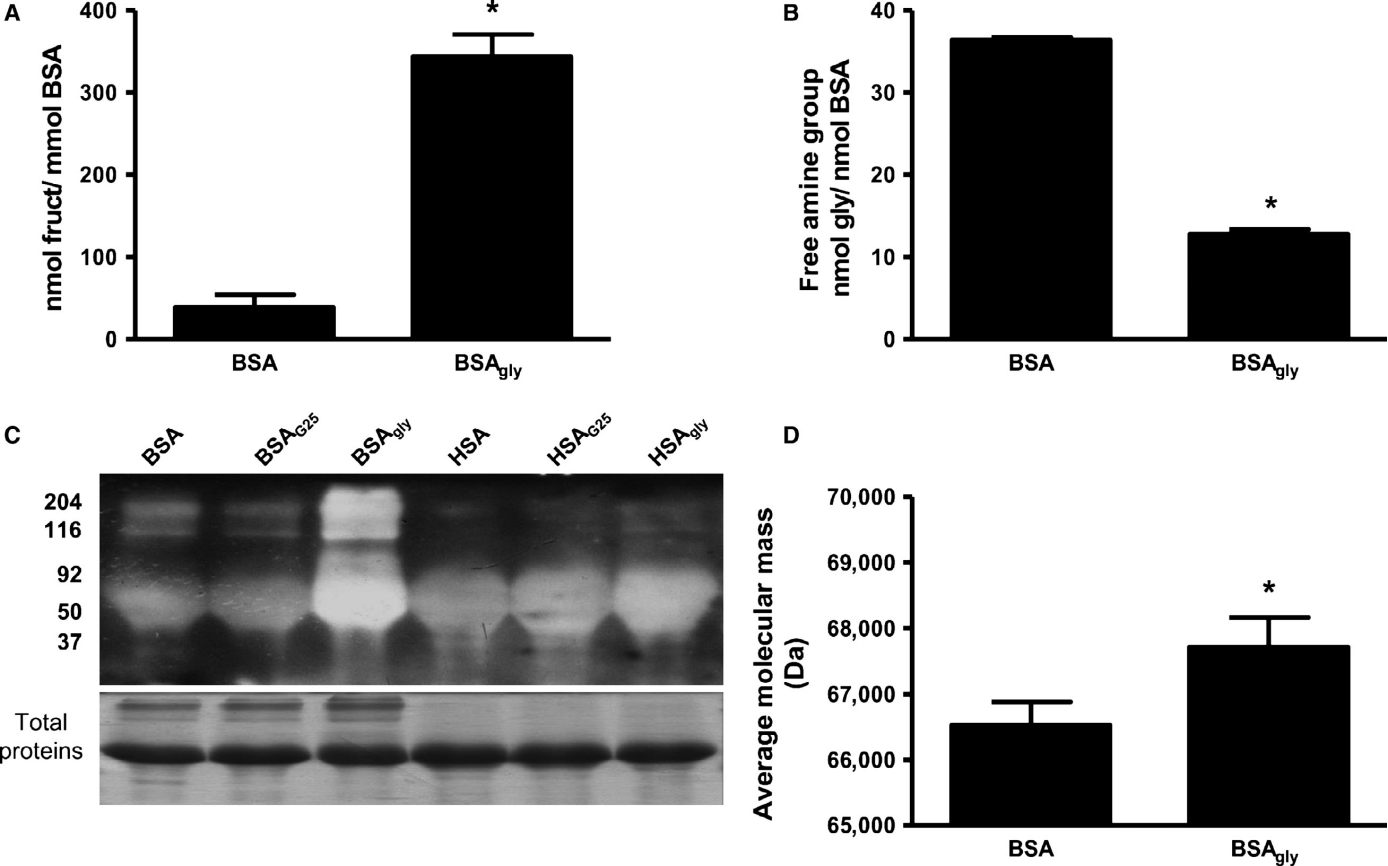
Validation of BSA glycation as indicated by the concentration of (A) fructosamine, (B) free amine and (C) levels of BSA and BSA_gly_ and (D) quantification of the protein mass of BSA and BSA_gly_. Values are expressed as mean ± SEM (n = 6). * *P* < 0.05 versus respective controls.

The initial experiments validated the success of BSA and HSA glycation with the high‐molecular‐weight AGEs. Figure 1 also shows that BSA glycation was more successful versus HSA glycation and therefore only BSA data are provided. BSA glycation is indicated by increased fructosamine levels in the BSA_gly_ versus BSA groups (343 ± 27 vs. 39 ± 15 nmol/mmol BSA, *P* < 0.05) (Fig. 1A). This result was mirrored by decreased levels of free amine in the BSA_gly_ group from 36 ± 1 to 12 ± 1 nmol/nmol BSA (*P* < 0.05 vs. native BSA, Fig. 1B). Furthermore, our data revealed increased molecular mass for BSA glycation versus the native BSA groups (*P* < 0.05, Fig. 1C and D).

### Impairment of cardiac contractile function under high glucose conditions and by BSA_gly_

**Figure 2:**
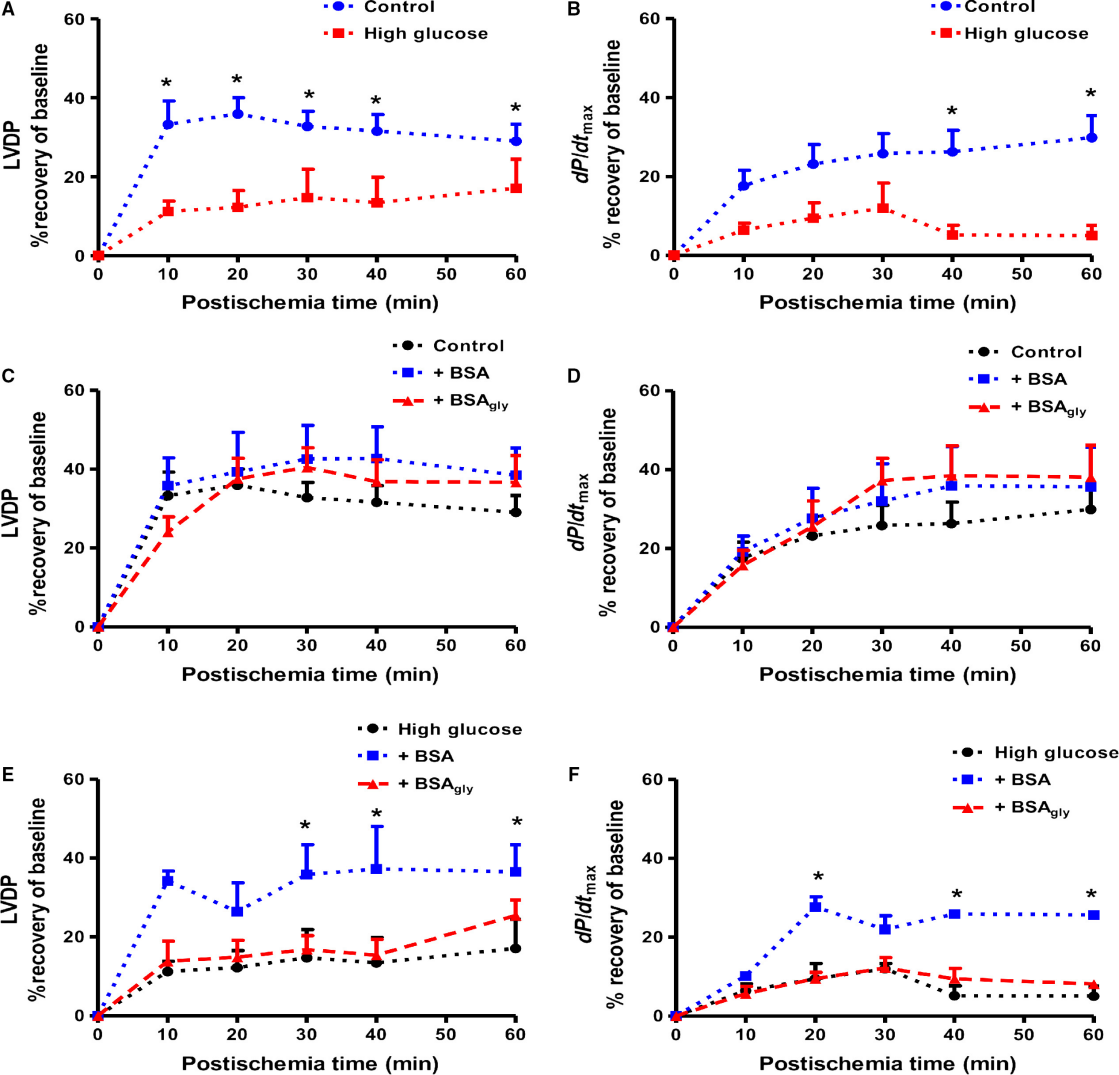
Cardiac contractile function following global ischemia and reperfusion as indicated by (A) % left ventricular developed pressure (LVDP) and (B) % dP/dt_max_ recovery under baseline and acute high glucose conditions; % LVDP recovery with native bovine serum albumin (BSA) versus glycated albumin (BSA_gly_) under (C) baseline and (D) acute high glucose conditions; % dP/dt_max_ recovery (E) under baseline and (F) acute high glucose conditions with native bovine serum albumin (BSA) versus glycated albumin (BSA_gly_). Values are expressed as mean ± SEM (*n* = 6). * *P* < 0.05 versus respective controls.

Hearts exposed to high glucose perfusate displayed decreased cardiac contractile function following ischemia‐reperfusion versus baseline glucose conditions. This was reflected by a significant decrease in the % LVDP recovery and dP/dt_max_ in the high glucose versus control groups (Fig. 2A and B). RPP followed the same trend, however, there were no differences in the heart rate and coronary flow rate (data not shown). Neither BSA nor BSA_gly_ treatment caused significant effects on heart function in response to ischemia‐reperfusion under control conditions (11 mmol/L glucose) (Fig. 2C and D). However, under high glucose conditions, BSA administration improved functional recovery versus BSA_gly_ with a % LVDP recovery of 36 ± 7 versus 25 ± 4 (*P* < 0.05), respectively, at the end of the reperfusion period (Fig. 2E). Likewise, the % dP/dt_max_ recovery was significantly improved with native BSA treatment versus BSA_gly_ (Fig. 2F).

### Infarct size and apoptosis are elevated in the BSA_gly_‐treated high glucose groups

**Figure 3:**
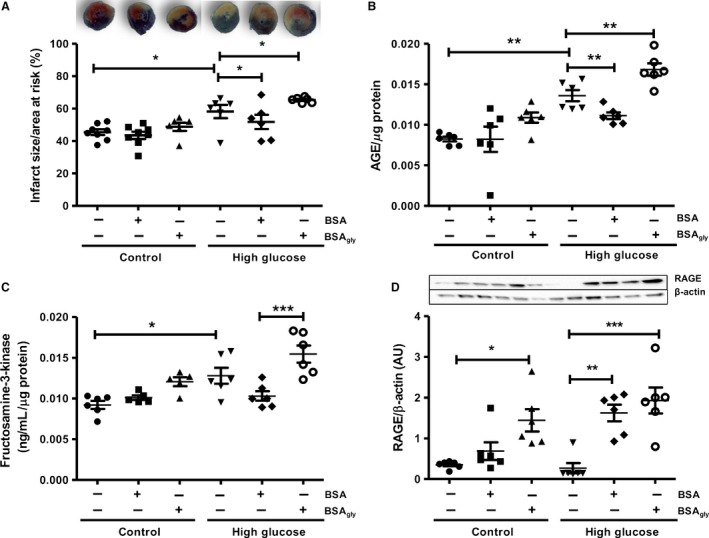
The effects of native bovine serum albumin (BSA) versus glycated albumin (BSA_gly_) on (A) infarct sizes (regional ischemia) and advanced glycation end product (AGE) pathway activation as indicated by: (B) AGE levels, (C) fructosamine‐3‐kinase (FN3K) levels, (D) receptor for advanced glycation end product (RAGE) expression. Values are expressed as mean ± SEM (*n* = 6). * *P* < 0.05, ** *P* < 0.01, *** *P* < 0.001 versus respective controls.

These data also revealed increased infarct size under high glucose perfusion conditions, that is from 45 ± 2% to 58 ± 4% (*P* < 0.05 vs. 11 mmol/L glucose control, Fig. 3A). As stated earlier, the administration of native BSA or BSA_gly_ did not result in any significant effect on infarct sizes for the control groups. By contrast, treatment with BSA decreased infarct size versus the untreated high glucose group under high glucose perfusion conditions (*P* < 0.05). However, this protective effect was lost with glycation as BSA_gly_ further increased infarct size to 65 ± 3% versus 58 ± 4% in the high glucose untreated group (*P* < 0.05, Fig. 3A).

### Myocardial AGE levels and markers of AGE metabolism are elevated by BSA_gly_ under high glucose perfusion conditions

We next assessed markers of myocardial AGE levels and metabolism as potential mediatory mechanisms that may play a role in the observed functional changes. Myocardial AGE formation was increased with ischemia‐reperfusion under high glucose versus controls (*P* < 0.05) (Fig. 3B). Moreover, BSA_gly_ administration resulted in a further elevation of AGE formation under these experimental conditions (*P* < 0.01), while native BSA attenuated AGE formation (*P* < 0.01) (Fig. 3B). Myocardial levels of FN3K were elevated by high glucose perfusions, while this was further increased by BSA_gly_ administration (*P* < 0.001 vs. BSA) (Fig. 3C). Protein expression of RAGE was increased by BSA_gly_ in the control glucose groups (*P* < 0.05 vs. control, Fig. 3D), while high glucose perfusions did not significantly alter these levels when compared to controls. In addition, high glucose hearts perfused with both native BSA and BSA_gly_, respectively, showed a significant increase in myocardial RAGE expression (Fig. 3D).

### Superoxide dismutase 1 and 2 expression is enhanced by native BSA perfusions in the high glucose setting

**Figure 4:**
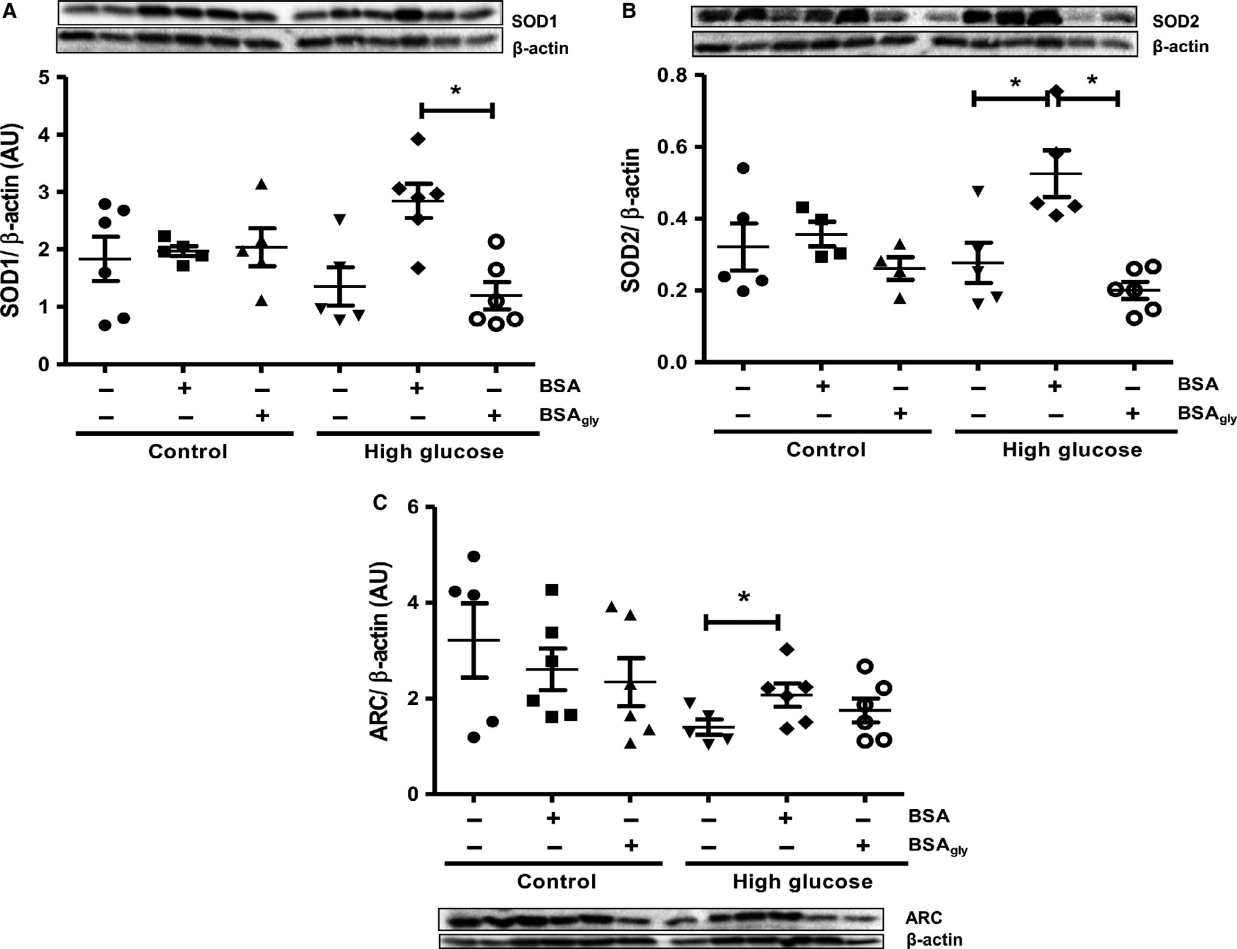
Albumin glycation following ischemia‐reperfusion under acute high glucose conditions results in decreased (A) superoxide dismutase (SOD1) and (B) SOD2 protein expression levels and also lower antiapoptotic (C) apoptosis repressor with caspase recruitment domain (ARC) protein levels. Values are expressed as mean ± SEM (*n* = 6). *P < 0.05 versus respective controls.

Tissue expression of SOD1 and SOD2 was unchanged with BSA and BSA_gly_ treatments, respectively, in the control and high glucose groups (Fig. 4A and B). The expression of both markers were, however, upregulated in the native BSA‐treated groups exposed to high glucose (*P* < 0.05 BSA vs. BSA_gly_ for SOD1; *P* < 0.05 BSA vs. high glucose and BSA_gly_, respectively, for SOD2). We also assessed myocardial apoptosis by evaluating ARC (an apoptosis inhibitor) levels and found an increase in BSA‐treated hearts under high glucose conditions (*P* < 0.05 vs. high glucose control, Fig. 4C). There were no significant changes in the control glucose groups, while perfusion with BSA_gly_ also did not affect ARC expression in the high glucose group.

## Discussion

This study revealed that the cardioprotective effect of native albumin is lost upon glycation in hearts exposed to ischemia‐reperfusion under (simulated) acute hyperglycemic conditions. In line with our previous findings, high glucose exposure *per se* resulted in impaired heart function and this was associated with larger infarct sizes, apoptosis, and increased myocardial AGE levels (Mapanga et al. 2014). Neither BSA nor BSA_gly_ elicited any effects on most of the experimental parameters measured under control glucose conditions, suggesting that such effects are only triggered in the context of high glucose availability.

Our data also show higher myocardial AGE levels when perfused hearts (exposed to high glucose) were treated with BSA_gly_. There are at least three possibilities whereby detrimental effects occur in this instance: (1) glycated albumin is still taken up by target cells but cannot exert its usual intracellular antioxidant role due to the structural modifications; and/or (2) glycated albumin binds to RAGE on target cells and trigger downstream effects to increase oxidative stress, AGE generation and inflammation; and/or (3) the glycation of BSA changes the binding capacity of this transporter protein, rendering it ineffective to carry circulating metabolites (Rondeau and Bourdon 2011). As this study revealed increased myocardial FN3K activity together with higher AGE levels under high glucose perfusion conditions, it supports the notion that downstream intracellular signaling is activated in this instance. In support, intracellular glycated proteins are physiological substrates of F3NK (Delpierre and Van Schaftingen 2003). RAGE expression was also increased under both control and high glucose experimental conditions with BSA_gly_ treatment, indicating this pathway likely involved in downstream damaging effects. However, others found that BSA glycation did not affect RAGE levels (Deluyker et al. 2016). The precise reasons for this remain unclear but it is likely due to variations in experimental protocols (in vivo vs. ex vivo); the duration of glycation exposure and the presence of ischemia and reperfusion. It is possible that RAGE may be involved in a temporal manner, that is active during the early stages following exposure to BSA glycation but that with prolonged exposure, there is a shift to RAGE‐independent pathways. However, these possibilities require further investigations to confirm its validity. Of note, cardiac RAGE levels showed no significant changes under high glucose conditions compared to controls. As AGE levels were increased with high glucose, a positive feedback effect would be predicted to also lead to higher RAGE levels. The reason for this finding remains unclear and requires further investigation with analysis of additional markers such as soluble RAGE (sRAGE) and high mobility group box 1 (HMGB1). It is also possible that we obtained these results since both ventricles were analyzed instead of only the ischemic tissue. Here, others found that RAGE upregulation occurs in the ischemic and not in the nonischemic remote zone (Bucciarelli et al. 2006).

We recently proposed that higher oxidative stress under hyperglycemic conditions (with ischemia‐reperfusion) may play a central role by triggering damaging downstream effects, that is increasing AGEs that can also fuel inflammation and apoptosis (Mapanga and Essop 2016). The current findings establish that BSA treatment under such experimental conditions offered cardioprotection by augmenting antioxidant capacity. The precise mechanism(s) whereby such changes occur remain unknown although it is likely that BSA exerts intracellular antioxidant effects as demonstrated by increased SOD1 and SOD2 expression levels. In support, albumin taken up by target cells can elicit antioxidant effects [reviewed in Francis (2010)]. However, it is unclear whether this is the case for cardiomyocytes, although albumin can be taken up by endothelial cells (Francis 2010). These same effects are, however, not observed under baseline glucose conditions and this possibly occurs as glycation levels are not high enough to cause significant oxidative stress to trigger SOD activation.

### Limitations of the study

It would have added additional insights if we evaluated additional markers that relate to the AGE‐RAGE pathway, for example, sRAGE. In addition, although we assessed expression of markers of oxidative stress, these data would have been supported by additional enzyme activity assays such as catalase and SOD, and also actual levels of various species of oxidative stress.

## Conclusions

In summary, this study established that glycation abolished albumin‐mediated cardio‐protection with ischemia‐reperfusion under high glucose conditions and this centers around the loss of antioxidant capacity and increased activation of the AGE pathway together with elevated apoptosis.

## Conflict of Interest

The authors hereby confirm that no conflict of interests and no competing financial interests exist.
